# Lateral Plate Versus Isolated Lag Screw Fixation of Unstable Distal Fibula Fractures: A Systematic Review

**DOI:** 10.1155/aort/9302062

**Published:** 2026-02-09

**Authors:** Aarohanan Raguragavan, Dujinthan Jayabalan, Rajitha Gunaratne

**Affiliations:** ^1^ Department of Orthopaedics, Fiona Stanley Hospital, Murdoch, Western Australia, Australia, fsh.health.wa.gov.au; ^2^ Medical School, The University of Western Australia, Nedlands, Western Australia, Australia, uwa.edu.au; ^3^ Sir Charles Gairdner Hospital, Nedlands, Western Australia, Australia, scgh.health.wa.gov.au; ^4^ Department of Orthopaedic Surgery, Joondalup Health Campus, Perth, Western Australia, Australia, joondaluphealthcampus.com.au; ^5^ Perth Orthopaedic Specialist Centre, Perth, Western Australia, Australia; ^6^ Curtin University, Perth, Western Australia, Australia, curtin.edu.au

**Keywords:** ankle fractures operative technique, ankle fractures postoperative outcomes, lateral plate fixation, unstable distal fibula ankle fracture, unstable lateral malleolus fracture

## Abstract

**Background:**

Lateral plate fixation, the current standard for fixation of unstable distal fibular fractures, can compromise the minimal soft tissue envelope resulting in poorer postoperative outcomes. Isolated lag screw fixation represents a novel method of distal fibula fixation.

**Methods:**

Two independent reviewers conducted a literature search of PubMed, EMBASE, Google Scholar and MEDLINE (Ovid) in February 2024. Randomised control trials (RCTs), non‐randomised control trials and cohort studies were included. Risk of bias was assessed with the ROBINS‐I tool. PRISMA guidelines were followed. This systematic review was prospectively registered in the PROSPERO register (CRD42024521746). Results were synthesised by structured qualitative analysis.

**Results:**

Four studies (207 patients) were included. Compared to lateral plate fixation, lag screw‐only fixation for distal fibula fractures resulted in reduced operative times, incision lengths, costs, hardware‐related issues, ankle symptomatology, and complications, with comparable or improved functional outcomes and similar radiographic healing under equivalent postoperative care. These findings were applicable to patients under 60 years old with noncomminuted, oblique, or spiral unstable distal fibula fractures.

**Conclusion:**

Isolated lag screw fixation represents a novel method of distal fibular fracture fixation in select patients. Reducing hardware burden on the soft tissue envelope improved operative and patient outcomes without compromising standard postoperative care. The methodological and clinical differences among the included studies limited this review. Additionally, only four eligible studies were included, all with significant bias.

## 1. Introduction

Accounting for 9% [1] of fractures in the adult population, ankle fractures are among the most common fractures treated by orthopaedic surgeons. Isolated distal fibula fractures account for 55% of all ankle fractures [2]. Although the consensus regarding functional outcomes following operative management of ankle fractures has been generally positive [3, 4], growing evidence exists to suggest that health outcomes and quality of life remain reduced for the short‐ and medium‐term periods compared to matched population norms [3, 4]. Complications from operative management of distal fibula fractures hinder improvements in health‐related quality of life [3].

The goal of operative management of fibula fractures is to ensure proper anatomic reduction, alignment, and fixation to restore normal ankle function [5], enabling congruity of the ankle mortise in all positions of dorsiflexion and plantarflexion [5]. Failure to address these issues is associated with poor outcomes and presages posttraumatic osteoarthritis [5].

The Arbeitsgemeinschaft fur Osteosynthesefragen (AO) technique utilising lateral fibula plate osteosynthesis to neutralise an interfragmentary compressive screw [6] has been common practice for over 50 years and has been the standard treatment for unstable distal fibula fractures [7]. The bulk of a fibula plate, however, may compromise the minimal soft tissue [8, 9] over the distal fibula, causing wound dehiscence, infection, or wound edge necrosis [9, 10]. Furthermore, protruding lateral plating is often symptomatic and may lead to increased rates of reoperation, altogether worsening postoperative outcomes [9].

Alternative nonoperative surgical techniques, fixation with lag screws in isolation, intramedullary and bio‐absorbable fixation [9] represent methods employed to reduce the added insult of surgery on the often already compromised soft tissue envelope [9]. By facilitating shorter incision lengths and reduced implant bulk, isolated lag screw fixation may reduce the risk of soft tissue complications whilst providing adequate stability [8, 9].

Isolated lag screw fixation represents a potential operative technique in select patient groups where congruency and stability can be ensured, whilst minimising the complications often associated with the widely utilised lateral plate technique. To date, no systematic reviews have been published on this topic. This systematic review aims to (i) summarise the literature and clarify strengths and weaknesses of current evidence on isolated lag screw fixation in comparison to lateral plate osteosynthesis with lag screw fixation; (ii) demonstrate whether there is a benefit to either isolated lag screw fixation or lateral plate osteosynthesis with lag screw fixation; and (iii) identify areas of need for future research.

## 2. Methods

The structure of this systematic review followed the Preferred Reporting Items for Systematic Reviews and Meta‐Analyses (PRISMA) guidelines (Supporting Tables 1 and 2) [11–13] and aligns with the described methodology [14]. This systematic review was prospectively registered in the PROSPERO register (CRD42024521746).

### 2.1. Key Outcomes Following Orthopaedic Surgery

There is importance in linking clinical variables with health‐related quality of life [15]. Focus must be held when conducting orthopaedic research to ensure measurement of biological and physiological variables, as well as symptoms, functional status, general health perceptions, and overall quality of life [16, 17]. As such, the data collection and representation in this systematic review aim to highlight the comparison of these outcomes in patients receiving either isolated lag screw fixation or lateral plate osteosynthesis with lag screw fixation.

### 2.2. Eligibility Criteria

Studies considered for review had the following characteristics: (i) patients younger than 60 years of age; (ii) patients with unstable distal fibular fractures, (iii) patients deemed suitable for operative management of distal fibular fractures; (iv) reporting differences in operative technique for both lateral plate osteosynthesis and isolated lag screw fixation; (v) reporting differences in postoperative management of lateral plate osteosynthesis compared to isolated lag screw fixation; and (vi) investigating postoperative outcomes of lag screw fixation only patients compared to lateral plate osteosynthesis patients. These studies were restricted according to the following report characteristics: (i) English language and (ii) original articles in peer‐reviewed journals.

Randomised‐control trials (RCTs), non‐randomised control trials and cohort studies were deemed suitable for inclusion. All clinical trials evaluating the effects of isolated lag screw fixation against lateral plate osteosynthesis on postoperative outcomes were included.

### 2.3. Information Sources and Search Strategy

**FIGURE 1:**
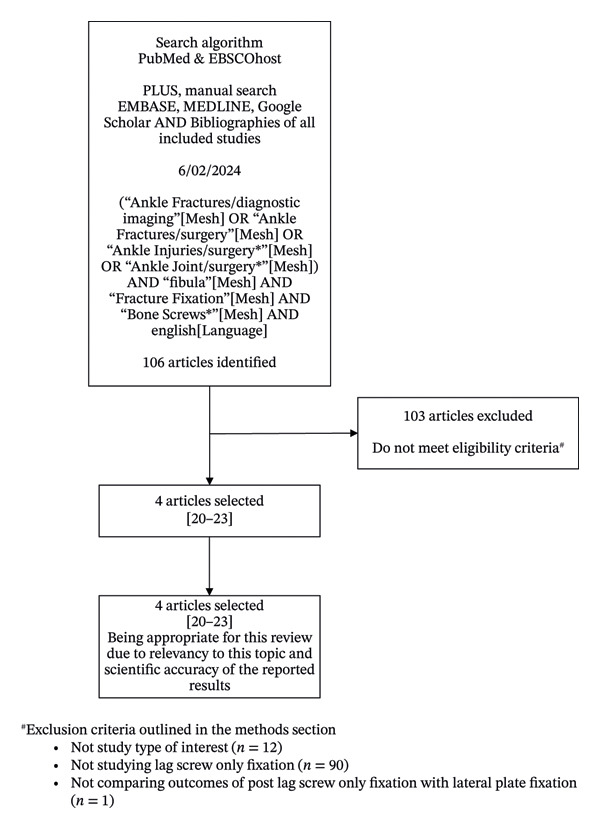
Search strategy used for systematic review of lateral plate versus lag screw only fixation in unstable distal fibula ankle fractures.

In February 2024, a preliminary literature search for all studies matching the eligibility criteria (Figure 1) was conducted independently by A.R. and R.G. using Medical Subject Headings (MeSH) keyword search with Boolean operators on PubMed and EMBASE. Additionally, a manual search of MEDLINE (Ovid) and Google Scholar and a review of the bibliographies of each included study were conducted to identify studies that were not retrieved in the preliminary MeSH keyword search. All identified articles were retrieved from the above databases.

### 2.4. Study Selection

Following the initial search, duplicates were manually removed, and A.R. and R.G. independently screened titles and abstracts after MeSH keyword and manual searches. A full text review of studies coded as included or potentially eligible was independently performed by A.R. and R.G. Studies were excluded if they did not meet eligibility criteria. Consensus for studies included for review was achieved by discussion between A.R., D.J., and R.G. based on the predetermined eligibility criteria. The study selection eligibility process is illustrated in a PRISMA flow diagram (Figure 1).

### 2.5. Data Items and Extraction

Three prepiloted data extraction forms were developed by A.R. Data items for assessment of study quality (Table 1), operative methods and postoperative management (Table 2), and study results (Table 3) were predetermined by A.R. and R.G. Data extraction were then performed by A.R. and D.J. independently using standardised pilot forms, with differences resolved by consensus between A.R., D.J., and R.G [22]. Results comparing the isolated lag screw and the lateral plate with the lag screw were organised for easier comparison, with recurring themes grouped together for discussion.

**TABLE 1:** Summary of study characteristics for included articles comparing lateral plate and lag screw fixation in unstable distal fibula fractures.

Year	Author	No. of patients ‐ Lag screw fixation ‐ Lateral plate fixation	Study design	Study outcomes measured		Participation rate—lag screw fixation—lateral plate fixation	Level of evidence	Patient demographics			
				Radiographic	Clinical			Selection criteria: age (mean)—lag screw fixation—lateral plate fixation	Sex—lag screw fixation—lateral plate fixation	Location	Fracture types included/selection:
2001	Tornetta et al. [19]	‐ 47 ‐ NR	Prospective	Presence of arthritis	Lateral pain complaints Palpable hardware Shoe wear restrictions Removal of metal Range of movement	‐ 42/47 (91%) ‐ NR	III	Patients older than 50 years of age were excluded. NR NR	NR NR	United States	Included fracture types: ‐ Simple trans‐syndesmotic with medial ligament injury ‐ Trans‐syndesmotic with medial malleolus fracture ‐ Simple suprasyndesmotic with medial malleolus fracture Inclusion criteria: ‐ Oblique fractures without comminution, long enough for two lag screws spaced, ≥ 1 cm apart

2006	McKenna et al. [20]	‐ 25 ‐ 25	Retrospective cohort	Quality of initial reduction	Foot and Ankle Outcomes Questionnaire Duration of lateral ankle pain Complications	‐ 18/25 (72%) ‐ 16/25 (64%)	III	16–55 (mean: 37.9) 14–59 (mean: 37.3)	80% M 68% M	Ireland	Lauge‐Hansen supination–external rotation IV ankle fractures ‐ Lateral malleolus only‐ ‐ Bi‐malleolar ‐ Tri‐malleolar Lag screw fixation criteria: Simple oblique or spiral fibula fractures without comminution, long enough for two screws placed 1 cm apart and perpendicular to the fracture line

2021	Paez et al. [21]	‐ 32 ‐ 51	Retrospective	Fracture healing at 6 weeks	FAAM SANE Patient satisfaction scores Return to sport Symptomatic implants Complication rates Implant removal Implant bothersome	‐ 45/83 (52%)	III	Adolescents less than 20 years of age. 11.6–18 (mean: 16.1) 12.1–19.6 (mean: 16.2)	72% M 47% M	United States	‐ Lateral malleolus only ‐ Bi‐malleolar ‐ Tri‐malleolar ‐ Weber B ‐ Weber C

2022	Grisdela Jr. et al. [22]	‐ 10 ‐ 17	Retrospective	Stable Mortise at 6 Weeks	Repeat surgery Infection Complication Clinical union at 6 weeks Other: Hospital cost	‐ NR	III	Patients over the age of 18. 48.4 (SD: 17.6) 52.4 (SD: 8.9)	30% M 35% M	United States	Included fractures: Lauge‐Hansen SER IV (Weber B) with bi‐malleolar injury or deltoid ligament disruption

*Note:* M, percentage male; No., number.

Abbreviations: FAAM, foot and ankle ability measure; NR, not reported; PR, participation rate; SANE, single assessment numeric evaluation; SD, standard deviation.

**TABLE 2:** Summary of operative results from studies comparing lateral plate and lag screw fixation in unstable distal fibula fractures.

**Year**	**Author**	**Follow-up**		**Operative**	**Weight-bearing status/immobilisation**	**Management of concomitant injuries**

2001	Tornetta et al. [19]	1.6 (years)	Lag screw	Incision: ‐ Curved A–P ‐ Length: 3 cm (30% shorter than plate fixation) Screw fixation: ‐ A–P ‐ Majority cortical lag screws ‐ Distal screws were occasionally partially threaded cancellous lag screws Fixation time: 13 min	‐ Short leg plaster cast for 6 weeks ‐ Weight‐bearing at 6 weeks if syndesmosis intact ‐ Weight‐bearing at 10–12 weeks if syndesmosis fixed	Syndesmotic injury: ‐ Syndesmotic screw placement Medial malleolar fracture: ‐ Standard ORIF Deltoid ligament injury: ‐ Treated closed
			Lateral plate	Incision: ‐ Length: NR (43% longer than lag screw fixation) Fixation time: NR	NR	

2006	McKenna et al. [20]	12 (7–26) (months)	Lag screw	Incision: ‐ Lateral incisions over the anterior edge of the fibula at the fracture site Screw fixation: ‐ A–P ‐ 2–3 stainless steel lag screws ‐ Partially threaded cancellous screws or 3.5 mm cortical screws in lag mode	‐ Below‐knee cast worn for 6 weeks ‐ Partial weight‐bearing was permitted at 4 weeks ‐ Full weight‐bearing at 8 weeks	Medial malleolar fracture: ‐ Standard screw fixation Deltoid ligament injury ‐ Treated closed, assuming reduced mortise
		13 (6–26) (months)	Lateral plate	Plate fixation: ‐ Neutralisation plate ‐ ≥ 1 lag screw compression	‐ Below‐knee cast worn for 6 weeks ‐ Partial weight‐bearing was permitted at 4 weeks ‐ Full weight‐bearing at 8 weeks	

2021	Paez et al. [21]	54 ± 25 (months)	Lag screw	Tourniquet time: −49 ± 17 min, *p* = 0.001 Incision: ‐ Standard approach, AO technique ‐ Length: 6 ± 1 cm, *p* = 0.008 Screw fixation: ‐ Standard AO technique ‐ A–P ‐ ≥ 2 screws lag screw insertion	Immobilisation time (wk): 5 ± 3, *p* = 0.371 WB restriction time (wk): 6 ± 1, *p* = 0.946 ‐ Splinted or cast for 2–6 weeks based on the surgeon’s preference ‐ Progressive weight‐bearing between 2 and 4 weeks ‐ Full weight‐bearing at 6 weeks (with confirmed radiographic healing) ‐ Physical therapy initiated at 6 weeks ‐ Return to sport at 3 months	Medial malleolar fracture: ‐ Screw fixation with 4 cm screw Posterior malleolar fracture: ‐ Posterolateral approach ‐ Screw fixation with 4 cm screw
		46 ± 32 (months)	Lateral plate	Tourniquet time: −64 ± 20 min, *p* = 0.001 Incision: ‐ Standard approach, AO technique ‐ Length: 8 ± 2 cm, *p* = 0.008 Plate fixation: ‐ Neutralisation plate −3 bicortical screws proximally ‐ Unicortical or bicortical screws distally ‐ ≥ 1 lag screw	Immobilisation time (wk): 6 ± 2, *p* = 0.371 WB restriction time (wk): 6 ± 1, *p* = 0.946 ‐ Splinted or cast for 2–6 weeks based on the surgeon’s preference ‐ Progressive weight‐bearing between 2 and 4 weeks ‐ Full weight‐bearing at 6 weeks (with confirmed radiographic healing) ‐ Physical therapy initiated at 6 weeks ‐ Return to sport at 3 months	

2022	Grisdela Jr. et al. [24]	130 (SD 89) (days)	Lag screw	Hospital costs mean ± SD: ‐ $592 ± $229, *p* < 0.0001 Screw fixation: ‐ Lag screw ‐ Proximal + distal fibula pro tibia screw fixation	‐ Early range of motion and protected weight‐bearing for 6 weeks ‐ Weight‐bearing as tolerated at 6 weeks	Syndesmotic injury: ‐Treated according to surgeon preferences
		143 (SD 63) (days)	Lateral plate	Hospital costs mean ± SD: $1950 ± $562, *p* < 0.0001 Plate fixation: ‐ Standard AO technique ‐ Neutralisation plate ‐ Lag screws	‐ Early range of motion and protected weight‐bearing for 6 weeks ‐ Weight‐bearing as tolerated at 6 weeks	

*Note:* A–P, anterior to posterior placement; AO, Arbeitsgemeinschaft fur Osteosynthesefragen; *p*, *p* value; wk, weeks.

Abbreviations: NR, not reported; ORIF, open reduction internal fixation; SD, standard deviation.

**TABLE 3:** Summary of postoperative results from studies comparing lateral plate and lag screw fixation in unstable distal fibula fractures.

**Year**	**Author**	**Follow-up duration**		**Clinical outcomes**	**Radiographical outcomes**

2001	Tornetta et al. [19]	1.6 years	Lag screw	Complaint of lateral pain: 2% (1/47) Palpable hardware: 0% (0/47) Shoe wear restriction: 0% (0/47) Removal of hardware: 0% (0/47) Range of motion: Dorsiflexion: 8° (0°–20°) Plantarflexion: 37° (20°–50°)	Radiographical signs of arthritis: 0% (0/47)
			Lateral plate	Complaint of lateral pain: 17% Palpable hardware: 56% Shoe wear restriction: 15% Removal of hardware: 31% Range of motion: Dorsiflexion: 8° (0°–20°) Plantarflexion: 37° (20°–50°)	Radiographical signs of arthritis: 0%

2006	McKenna et al. [20]		Lag screw	Palpable metalware: 1/18 (5%), *p* = 0.015 Additional surgery: 0/18 (0%), *p* = 0.023 Infection: 0/18 (0%), *p* < 0.001 AOFAS—core standardised mean score: 86, *p* = 0.02 AOFAS—shoe comfort standardised score: 74, *p* = 0.211 Duration of pain symptoms: < 3 months, *p* = 0.004	All lateral malleolar fractures united with an anatomically reduced ankle mortise
			Lateral plate	Palpable metalware: 8/16 (50%), *p* = 0.015 Additional surgery: 5/25 (20%), *p* = 0.023 Infection: 4/25 (16%), *p* < 0.001 AOFAS—core standardised mean score: 76, *p* = 0.02 AOFAS—shoe comfort standardised score: 60, *p* = 0.211 Duration of pain symptoms: > 6 months, *p* = 0.004	All lateral malleolar fractures united with an anatomically reduced ankle mortise

2021	Paez et al. [21]	54 ± 25	Lag screw	SANE score: 92 ± 8, *p* = 0.361 FAAM—ADL: 98.2 ± 3.1, *p* = 0.36 FAAM—sports: 93.2 ± 11.0, *p* = 0.746 Complications: 1/32, *p* = 0.644 Satisfaction 0–10 (n): 8 (3), 9 (2), 10 (14), *p* < 0.329 Return to all activities sports (n): 18/19, *p* = 0.607 Function (*n*): normal (14), nearly normal (5), *p* = 0.333 Implant bothersome (*n*): 5/15, *p* = 0.044 Implant removed: 4/28, *p* = 0.386	100% healing rates without loss of reduction
		46 ± 32	Lateral plate	SANE score: 93 ± 11, *p* = 0.361 FAAM—ADL: 98.1 ± 2.1, *p* = 0.36 FAAM—sports: 94.0 ± 8.2, *p* = 0.746 Complications: 4/47, *p* = 0.644 Satisfaction 0 to 10 (n): 8 (1), 9 (5), 10 (15), *p* = 0.329 Return to all activities sports (n): 18/21, *p* = 0.607 Function (*n*): normal (12), nearly normal (9), *p* = 0.333 Implant bothersome (*n*): 15/27, *p* = 0.044 Implant removed: 11/40, *p* = 0.386	100% healing rates without loss of reduction

2022	Grisdela Jr. et al. [24]		Lag screw	Repeat surgery: 50%, *p* = 0.012 Infection: 0%, *p* = 1 Complication: 60%, *p* = 0.002 ‐ Removal of syndesmotic screw (*n*): 4 ‐ Removal of Lisfranc screw (*n*): 1 ‐ Removal of fibula fixation (*n*): 0 ‐ Postoperative deep vein thrombosis (*n*): 1 Clinical union at 6 weeks: 100%, *p* = 1	Stable mortise at 6 weeks: 100%, *p* = 1
			Lateral plate	Repeat surgery: 18%, *p* = 0.012 Infection: 0%, *p* = 1 Complication: 12%, *p* = 0.002 ‐ Removal of syndesmotic screw (*n*): 2 ‐ Removal of Lisfranc screw (*n*): 0 ‐ Removal of fibula fixation (*n*): 1 ‐ Postoperative deep vein thrombosis (*n*): 0 Clinical union at 6 weeks: 100%, *p* = 1	Stable mortise at 6 weeks: 100%, *p* = 1

*Note: n*, number; *p*, *p* value.

Abbreviations: ADL, activities of daily living; AOFAS, American Orthopaedic Foot & Ankle Society; FAAM, foot and ankle ability measure; SANE, single assessment numeric evaluation.

Variables collected were year, author, number of patients, study design, study outcomes measured, participation rate, level of evidence, patient demographics, age, sex, location of study, fracture types included, follow‐up, operative technique (incision length, tourniquet time and screw fixation), weight‐bearing status/immobilisation, management of concomitant injuries, clinical outcomes and radiographical outcomes.

### 2.6. Synthesis of Results

Qualitative analysis was performed by structured qualitative analysis with respect to pre‐existing guidelines [14].

### 2.7. Risk of Bias

**FIGURE 2:**
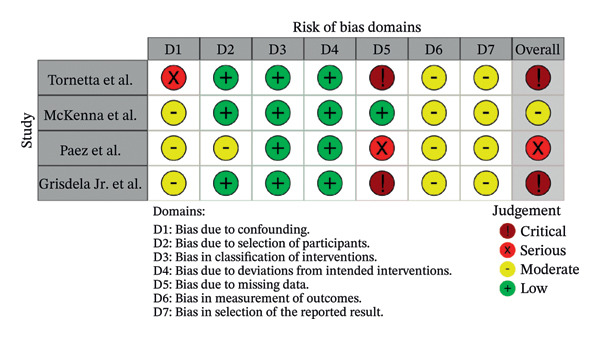
ROBINS‐I risk of bias by domain.

**FIGURE 3:**
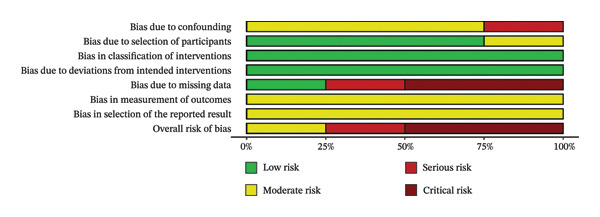
ROBINS‐I summary.

Qualitative analysis based on study quality was used to assess the risk of bias in individual studies with data tabulated in Table 1. ROBINS‐I (Risk of Bias in Non‐randomised Studies of Interventions, previously known as ACROBAT‐NRSI) (Figures 2 and 3.), was used to assess bias within each study. Meta‐analysis was precluded by clinical heterogeneity, variability in outcome measures, small study numbers and high risk of bias. Each study was assessed for significant selection, performance, detection or reporting bias, supported by the Cochrane guidelines on systematic reviews [23]. Assessment of bias was performed according to the PRISMA guidelines [11–13]. Levels of evidence for individual studies were assessed using previously outlined guidelines (NHMRC Evidence Hierarchy) [18]. Risk of bias assessment was performed by A.R. and D.J. independently, with disagreements resolved by consensus between A.R., D.J. and R.G.

## 3. Results

### 3.1. Study Selection

Following a detailed literature search, four studies were chosen to be included in this systematic review (Supporting Table 3). The studies demonstrated significant diversity between comparison groups, investigated a wide variety of postoperative outcomes, lacked preoperative data, and occasionally did not express data as mean ± standard deviation. The lack of clinical, statistical, and methodological homogeneity precluded meta‐analysis and direct comparison.

### 3.2. Study Characteristics and Risk of Bias Within Studies

To minimise reporting bias, a systematic review of all literature meeting the eligibility criteria (Figure 1) was conducted. Strength of evidence was determined by systematic analysis of the included studies (Figures 2 and 3) as well as through the use of validated tools. Risk of bias was most pronounced in Domain 5 of the ROBINS‐I tool, indicating risk due to missing data, thus implying that outcome estimates may be biased by incomplete follow‐up and selective reporting, limiting the reliability of the reported effect sizes. Variability of investigated outcomes and heterogeneity in data presentation precluded detailed and direct comparison of the included studies. Additionally, comparison of results was limited by irregularities in study design and statistical analyses.

Three studies [19–21] reported participation rates which ranged between 52% and 91%. One study did not report participation rates [24]. Low response rates may skew data more positively, as patients who are unable to respond may tend to be more unwell [25]. All studies reported follow‐up duration; length of follow‐up ranged between 129.7 days [24] and 4.5 years [21].

Study design was variable; three studies were retrospective [20, 21, 24], of which one was a cohort study [20]. One study was a prospective cohort study [19]. Investigated population ages were patients below 50 years of age [19], patients aged 14 to 59 [20], adolescents aged 11.6–19.6 [21], and adults over the age of 18 [24].

### 3.3. Operative Management of Distal Fibular Fractures

The primary fracture type for investigation across all studies was variations of noncomminuted, oblique, or spiral distal fibular fractures, classified using one or more of the AO [19], Weber [21, 24], or Lauge‐Hansen [20, 24] classifications.

A previous study identified that the incision used for isolated lag screw fixation was 30% shorter than that of the lateral plate fixation; additionally, the average time for fixation was reduced and reported as 13 min [19]. Similarly, another included study [21] reports that the length of incision and duration of tourniquet time were significantly (*p* < 0.05) shorter for patients undergoing isolated lag screw fixation.

All studies [19–21, 24] report using an anterior‐to‐posterior (A–P) technique of screw insertion for isolated lag screw fixation. Additionally, regarding isolated lag screw fixation, all studies [19–21, 24] suggested the use of at least two to three cortical lag screws and occasionally a partially threaded cancellous lag screw distally [19]. The three studies [20, 21, 24] reporting the operative technique of lateral plate with lag screw fixation utilised neutralisation plates with the use of at least one lag screw for compression.

Patients with concomitant medial malleolar fractures, posterior malleolar fractures, tri‐malleolar fractures, deltoid ligament injuries, and syndesmosis injuries were included in all studies [19–21, 24]. The treatment of associated ankle injuries was guided by surgeon preference and managed as standard [19–21, 24].

The operative costs of isolated lag screw fixation were significantly (*p* < 0.05) less than lateral plate fixation at $1949.97 for lateral plate fixation with lag screw and $592 for isolated lag screw fixation [24].

### 3.4. Postoperative Management of Distal Fibula Fractures

Three studies [20, 21, 24] utilised the same postoperative instructions for both lateral plate fixation and isolated lag screw fixation cohorts; one study [19] did not report postoperative instructions for their lateral plate fixation cohort. Immobilisation time with either a splint [21] or cast [19–21] varied between two [21] and 6 weeks [19–21]. Timing of return to weight‐bearing differed between studies, either opting for early range of motion and protected weight‐bearing at less than 6 weeks [24], progressive weight‐bearing between 2 and 4 weeks [21] or up to weight‐bearing at 10–12 weeks with syndesmosis fixation [19].

### 3.5. Outcomes Following Distal Fibula Fixation

Symptoms including complaints of palpable metalware (0% vs. 56%) [19, 20], duration of symptoms [20], and bothersome implants (33% vs. 56%) [21] were significantly (*p* < 0.05) lower in patients who had undergone isolated lag screw fixation. Similarly, lateral pain (2% vs. 17%) [19] and shoe wear restriction (0% vs. 15%) [19] were shown to occur at lower rates in patients who had undergone isolated lag screw fixation. Return to all activities in sport, satisfaction, and regaining normal or nearly normal function showed no difference between operative types [21]. Range of motion was identical between isolated lag screw fixation and lateral plate fixation cohorts [19].

One included study reported infection rates or additional surgery rates to be significantly higher (*p* < 0.05) in the lateral plate fixation cohort (0% vs. 20%) [20]. Contrastingly, one retrospective analysis found that reported reoperation rates were significantly (*p* < 0.05) higher in the lag screw fixation cohort (50% vs. 18%); however, when comparing reasons for reoperation, removal of the specific fibula fixation device accounted for one case in the lateral plate fixation cohort and zero cases in the isolated lag screw fixation cohort [24]. This study did not describe baseline differences between cohorts or explain why syndesmotic fixation was more frequently performed in the lag screw only group [24]. The same study also reported no significant difference in infection rates [24].

The studies reported 100% healing rates without loss of reduction [21], no radiological signs of arthritis [19], 100% stable mortise at 6 weeks [24], and distal fibula fractures united with an anatomically reduced ankle mortise [20].

There was no significant difference between the American Orthopaedic Foot & Ankle Society—Shoe Comfort Standardized Score [20], the Single Assessment Numerical Evaluation score [21], or the Foot and Ankle Ability Measure—Activities of Daily Living or sports section score [21]. Patients in the isolated lag screw fixation cohort reported significantly (*p* < 0.05) higher scores in the American Orthopaedic Foot & Ankle Society—Core Standardised Mean Score (86 vs. 76) [20].

## 4. Discussion

### 4.1. Summary of Evidence and Interpretation

The key findings of this systematic review were that, when compared to lateral plate fixation, isolated lag screw fixation of distal fibula fractures resulted in (i) reduced operative times, incision lengths, and operative costs; (ii) decreased rates of hardware and ankle symptomatology; (iii) lower complication rates; (iv) comparable or improved functional outcome scores; (v) equivalent postoperative instructions; and (vi) demonstrating identical rates of radiologic healing. The results of this systematic review apply to patients younger than 60 years of age with noncomminuted, oblique, or spiral, unstable distal fibula fractures.

### 4.2. Management of Distal Fibula Fractures

Studies included in this systematic review reveal that patients undergoing isolated lag screw fixation experience significantly reduced operative times [19, 21] and shorter incision lengths [19, 21]. Reduced operative times have been shown to reduce the risk of surgical site infections [26]. Shorter incision lengths decrease the insult of soft tissue damage and the ‘second hit’ of surgery [20]. Additionally, avoiding the use of bulky dynamic compression plates reduces compromise to the minimal soft tissue envelope of the distal fibula.

Where included, all surgeons opted for A–P insertion of their lag screws. Interestingly, a seminal study from 1989 investigating isolated lag screw fixation utilised a posterior‐to‐anterior (P–A) technique [27]. A further review similarly recommends P–A insertion of lag screws for interfragmentary compression; following a direct lateral approach to the fibula, a lag screw is countersunk and inserted P–A to compress the fracture [28]. Proposed benefits of P–A insertion include greater purchase in the deep cross‐sectional area of the anterior cortex [28] and countersinking of the screw in the flatter posterior cortex, enabling more even spread of stress [28] and reducing irritation to the peroneal tendon [28].

The importance of the fibula as an ankle stabiliser and the biomechanical stability of a lateral plate [8] are cited as reasons for surgeons’ apprehension when opting for isolated lag screw fixation [29]. However, throughout the follow‐up period of over 4 years in one study, no patient was seen to have developed instability of their ankle joint [21]. Isolated lag screw fixation was evaluated in a recent biomechanical study and was found to be non‐inferior to lateral plate fixation when at least two lag screws were placed [29] in tests of rotation and rotation to failure, with three lag screws showing no significant difference in the aforementioned tests as well as lateral bend [29].

Postoperative management, especially joint rehabilitation, determines functional outcome and thereby return to sports, work, and normal daily activities [30]. Postoperative management varied between studies. Studies opted for plaster casting or splinting, with immobilisation time between two and six weeks [19–21, 24], based on surgeon preferences and fracture healing. Progressive weight‐bearing instructions and return to full weight‐bearing also varied between less than six and up to 12 weeks depending on factors including signs of radiographical healing, repair of syndesmotic injury, and surgeon preferences. Importantly, there was no significant difference between weight‐bearing status, immobilisation time, or choice of immobilisation for patients in either the isolated lag screw or lateral plate cohort.

A consideration of a fixation method with lower absolute rigidity is the duration of immobilisation time and the complications associated with increased periods of immobilisation. However, the quality of evidence is poor, and short‐term complications are unclear [31], with longer‐term outcomes showing no observable difference [31]. Additionally, early ankle movement has been shown to increase the rates of fixation failure, surgical site infections, and metalwork removal [31]. Patients undergoing isolated lag screw fixation experienced similar or improved outcomes despite comparable postoperative treatment.

### 4.3. Postoperative Outcomes Following Isolated Lag Screw Fixation

Postoperatively, patients who underwent isolated lag screw fixation proved to have significantly better symptomatic outcomes [20, 21]. When compared to patients undergoing lateral plate fixation, those who underwent isolated lag screw fixation complained of less lateral pain, palpable hardware, and shoe wear restriction [19–21]. The isolated lag screw cohort reported equal levels of satisfaction, whilst finding their implant significantly less bothersome and the duration of pain experienced significantly shorter [20, 21].

Isolated lag screw patients’ functional outcome scores were either equal to or greater than those in the lateral plate fixation cohort [20, 21]. Reoperation rates and complications, including infection, were either equal or significantly lower [20] in all but one study [24], where patients were treated with two additional pro‐tibia screws, and resultingly, demand for syndesmotic screw removals increased the rates of reoperation (50% vs. 18%). Syndesmotic fixation was determined intra‐operatively, and all such cases underwent planned screw removal. Thus, the increased reoperation rate reflects surgeon decision‐making regarding syndesmotic stability rather than failure of the lag screw‐only construct. As treatment was delivered by a single surgeon, the exact cause of this discrepancy cannot be determined; however, it may be hypothesised that assessment of tibiofibular stability is more difficult when using a lag screw‐only construct.

All studies identified identical radiological reduction, stability, and healing rates between both lateral plate fixation and isolated lag screw fixation cohorts [19–21, 24]. Throughout follow‐up there were no radiological signs of arthritis in either cohort [19].

The protrusion and bulk of lateral fibula plating have been cited as a detriment to the minimal soft tissue envelope of the distal fibula [8], worsening symptomatology and increasing infection and reoperation rates. The findings of this systematic review are in keeping with this sentiment, revealing that isolated lag screw fixation reduced the bulk of fixation whilst maintaining biomechanical stability.

### 4.4. Review Limitations

The methodological and clinical heterogeneity of included studies limited this systematic review. Furthermore, only four studies met the eligibility criteria for inclusion, and these studies retain significant bias. However, this review represents the only evidence available thus far and reflects the state of current available data. Thus, this systematic review adds to the literature for operative fixation patients younger than 60 years of age with noncomminuted, oblique, or spiral, unstable distal fibula fractures. Future research should comprise adequately powered randomised controlled trials with clearly defined patient selection criteria, standardised surgical techniques, and uniform postoperative rehabilitation protocols. Such studies should incorporate patient‐reported outcome measures, cost‐effectiveness analyses, and long‐term follow‐up to evaluate the durability of fixation and the incidence of post‐traumatic osteoarthritis.

Variations in age, surgical expertise, cohort size, and operative and postoperative management impact the clinical picture; as such, literature evaluating the outcomes of isolated lag screw fixation may be inconsistent. Due to the clinical heterogeneity of the studies included, the potential for significant bias should be accounted for in the interpretation of these findings.

## 5. Conclusion

The authors recommend lag screw fixation for patients younger than 60 years of age with noncomminuted, oblique, or spiral, unstable distal fibula fractures in whom at least two lag screws can be placed. Patients are shown to benefit from reduced duration of pain and symptoms of irritability and palpable metalware, and equal or greater reported functional outcomes. Postoperative care does not need to be altered for patients to experience similar biomechanical stability, whilst infection and reoperation rates are shown to improve with isolated lag screw fixation.

## Author Contributions

Study conception and design: Aarohanan Raguragavan and Rajitha Gunaratne; data collection: Aarohanan Raguragavan and Dujinthan Jayabalan; analysis and interpretation of results: Aarohanan Raguragavan and Rajitha Gunaratne; draft manuscript preparation: Aarohanan Raguragavan.

## Funding

This research received no funding. Open access publishing was facilitated by The University of Western Australia, as part of the Wiley–The University of Western Australia agreement via the Council of Australian University Librarians.

## Disclosure

All authors reviewed the results and approved the final version of the manuscript.

## Conflicts of Interest

The authors declare no conflicts of interest.

## Supporting Information

Additional supporting information can be found online in the Supporting Information section.

## Supporting information


**Supporting Information 1** Supporting Table 1: PRISMA 2020 Checklist for Systematic Reviews.

**Supporting Information 2** Supporting Table 2: PRISMA 2020 Checklist for Abstracts of Systematic Reviews.

**Supporting Information 3** Supporting Table 3: Searching inputs used for PubMed and EMBASE for the systematic literature search.

## Data Availability

The data that support the findings of this study are available in the supporting information of this article. Any additional data, analytic methods, and study materials will be made available upon reasonable request from the corresponding author.
