# Supplementary material for: Lateral Plate Versus Isolated Lag Screw Fixation of Unstable Distal Fibula Fractures: A Systematic Review
Source: Adv Orthop. 2026 Feb 9;2026:9302062. doi: 10.1155/aort/9302062 (PMC13291888; doi:10.1155/aort/9302062)
Supplement: Supplementary file 3 — Supporting Information 3 Supporting Table 3: Searching inputs used for PubMed and EMBASE for the systematic literature search. [file AORT-2026-9302062-s001.docx]

**Supplementary Table 3: Searching inputs used for PubMed and EMBASE for the systematic literature search**

| **Database** |  | **Search Terms** |
| --- | --- | --- |
| PubMed (from inception to 6^th^ February 2024) | 1 | “Ankle Fractures/diagnostic imaging"[Mesh] OR "Ankle Fractures/surgery"[Mesh] OR "Ankle Injuries/surgery*"[Mesh] OR "Ankle Joint/surgery*”[Mesh] |
|  | 2 | "fibula"[Mesh] |
|  | 3 | "Fracture Fixation"[Mesh] |
|  | 4 | "Bone Screws*"[Mesh] |
|  | 5 | english[Language] |
| EMBASE (from inception to 6^th^ February 2024) | 1 | ("lateral malleolus fracture" OR "ankle fracture" OR "fibula fracture") |
|  | 2 | ("fixation" OR "fracture fixation") |
|  | 3 | ("lateral plate fixation" OR "plate fixation" OR "plating") |
|  | 4 | ("lag screw" OR "lag screw fixation") |
|  | 5 | #1 AND #2 AND (#3 OR #4) |
|  | 6 | LIMITS: English language, Human subjects |
